# Physicochemical and Pharmacokinetic Parameters in Drug Selection and Loading for Transdermal Drug Delivery

**DOI:** 10.4103/0250-474X.40340

**Published:** 2008

**Authors:** N. S. Chandrashekar, R. H. Shobha Rani

**Affiliations:** Department of Pharmaceutics, Al-Ameen College of Pharmacy, Hosur Road, Bangalore - 560 027, India

**Keywords:** Transdermal delivery, Physicochemical, Pharmacokinetics

## Abstract

Skin of an average adult body covers a surface of approximately 2 m2 and receives about one-third of the blood circulating through the body. The transdermal route of administration cannot be employed for a large number of drugs. The rationality of drug selection based on pharmacokinetic parameters and physicochemical properties of the drug are the important factors to be considered for deciding its suitability of drug for delivery by transdermal route.

Over the past three decades, developing controlled drug delivery has become increasingly important in the pharmaceutical industry. The pharmacological response, both the desired therapeutic effect and the undesired adverse effect, of a drug is dependent on the concentration of the drug at the site of action, which in turn depends upon the dosage form and the extent of absorption of the drug at the site of action. Skin of an average adult body covers a surface of approximately 2 m^2^ and receives about one-third of the blood circulating through the body. Skin contains an uppermost layer, epidermis which has morphologically distinct regions; basal layer, spiny layer, stratum granulosum and upper most stratum corneum, it consists of highly cornified (dead) cells embedded in a continuous matrix of lipid membranous sheets. These extracellular membranes are unique in their compositions and are composed of ceramides, cholesterol and free fatty acids. The human skin surface is known to contain, on an average, 10-70 hair follicles and 200-250 sweat ducts on every square centimeters of the skin area^1^. It is one of the most readily accessible organs of the human body. The potential of using the intact skin as the port of drug administration to the human body has been recognised for several decades, but skin is a very difficult barrier to the ingress of materials allowing only small quantities of a drug to penetrate over a period of time^2^.

The transdermal route of administration cannot be employed for a large number of drugs. The objective of this paper is to focus on the rationality of drug selection based on pharmacokinetic parameters and physicochemical properties of the drug. Physiochemical factors such as solubility, crystallinity, molecular weight <400, polarity, melting point <200, partition coefficient Log P (octanol-water) between −1.0 to 4 must be considered^3^ (Table 1). Biological factor should also be considered such as skin irritation, site of application of the patch e.g. scopolamine patch for motion sickness is applied backside of the ear and Transderm-Nitro is applied on the chest. When a pharmacologically active material has to be presented to the skin, an occlusive or allergic response is significant, limits have to be determined for the acceptability of the undesired effect^4^. The pharmacokinetic information of the drug is a critical factor in deciding its suitability for delivery by the transdermal route as it is suitable only for drugs whose daily dose is in few milligrams. The resulting plasma concentration of active agent depends on the clearance; however, if one assumes a small volume of distribution and relatively long half-life, plasma level in excess of few micrograms per milliliter is very unlikely. Another important factor is the half-life, (e.g., nitroglycerin t_1/2_ is 3 min) which provides information on the disposition of a drug in our body other parameters such as effective plasma level; also determine whether a transdermal delivery can be developed or not (Table 2).

**TABLE 1:** FACTORS TO BE CONSIDERED FOR TRANSDERMAL DOSE CALCULATION

Physiochemical	Pharmacokinetic	Biological
Solubility Crystallinity Molecular weight Polarity Melting point	Half-life Volume of distribution Total body clearance Therapeutic plasma concentration Bioavailable factor	Skin toxicity Site of application Allergic reactions Skin metabolism Skin permeability

**TABLE 2:** IDEAL PROPERTIES OF DRUG CANDIDATE FOR TRANSDERMAL DRUG DELIVERY

Parameter	Properties
Dose	Should be low (<20 mg/day)
Half-life in h	10 or less
Molecular weight	<400
Partition coefficient	Log P (octanol-water) between-1.0 and 4
Skin permeability coefficient	>0.5 × 10^−3^ cm/h
Skin reaction	Non irritating and non-sensitizing
Oral bioavailability	Low
Therapeutic index	Low

One can estimate the skin input rate of a drug required from its transdermal system based on volume of distribution (Vd), total body clearance (Cl_T_) and steady state or therapeutic concentration (CPss) under steady state conditions, the drug input rate from its transdermal system is expected to be equal to its output rate, determined by total body clearance multiplied by the therapeutic plasma concentration. This relationship can be expressed using following mass balance equation; input rate = dosing rate × bioavaliable factor (F), output rate = total body clearance × steady state plasma concentration and input rate = output rate or F × dosing rate = CL_T_×CPss….1, where CL_T_ is total body clearance, CPss is average target plasma concentration. Since epidermis is metabolically inert, F = 1. For most drug compounds, total body clearance is the product of volume of distribution and total elimination rate (K_E_), CL_T_ = K_E_ × V_d_….2. Thus the required flux from a transdermal patch can be calculated by normalizing the dosing rate Eqn. 1 for the surface area (A, cm^2^); Flux, Jss = CL_T_ × CPss/A…3.

Here is an example to determine the feasibility of the anticonvulsant drug, primidone for 10 cm^2^ transdermal patch, currently administered 750 mg/day as a tablet. The required flux (Jss) or input rate can be calculated from the pharmacokinetic properties of the drug, therapeutic concentration 10 μg/ml, total body clearance and elimination half-life which was determined to be 0.78 ml/kg/min and 4 h, respectively, permeability coefficient is 5 × 10^−3^ and saturation solubility is 1 mg/ml^5^.

Since the drug is absorbed completely, F = 1, the required output rate of primidone can be calculated from Eqn. 3; CL = 0.78 ml/kg/min × 70 kg (normal body weight) = 54.6 ml/min = 3276 ml/h. Out put rate = (3276 ml/h×10 μg/h) ÷ 0 cm^2^ = 3276 μg/cm^2^/h. The input rate, transdermal flux in this case, is 3.3 mg/cm^2^/h is required from the transdermal patch of primidone. The mass of drug that can be delivered across the skin is M = P_estimate_ × Cs = 5×10^−3^×1 = 5 μg/cm^2^/h the area of the patch required to deliver therapeutic plasma level of primidone is Jss ÷ M = 3300 μg/h ÷ 5 μg/cm^2^/h = 660 cm^2^

From the above calculations, it is seen that a large area of the body viz. 600 cm^2^ is required to deliver the therapeutic dose of primidone from the transdermal patch which is not desirable. Thus transdermal patch of 10 cm^2^ would not be feasible for primidone. This paper makes an attempt to give information about the suitability of the drug(s) for the transdermal drug delivery systems based on their physiochemical and pharmacokinetic parameters.
